# Predictors of 15-Day Survival for the Intensive Care Unit Patient on Continuous Renal Replacement Therapy: A Retrospective Analysis

**DOI:** 10.7759/cureus.8175

**Published:** 2020-05-18

**Authors:** Abdul Hasan Siddiqui, Gautam Valecha, Jwalant Modi, Amina Saqib, Chanudi Weerasinghe, Faraz Siddiqui, Suzanne El Sayegh

**Affiliations:** 1 Pulmonary and Critical Care Medicine, University of Illinois Urbana Champaign, Champaign, USA; 2 Hematology-Oncology, Staten Island University Hospital, Staten Island, USA; 3 Nephrology, University of Cincinnati College of Medicine, Cincinnati, USA; 4 Pulmonary/Critical Care, Robert Wood Johnson Hospital, New Brunswick, USA; 5 Internal Medicine, Staten Island University Hospital, Staten Island, USA; 6 Pulmonary and Critical Care Medicine, Robert Packer Hospital, Sayre, USA; 7 Internal Medicine, Zucker School of Medicine at Hofstra Northwell, Staten Island University Hospital Northwell Health, Staten Island, USA

**Keywords:** continuous renal replacement therapy (crrt), cvvh, medical icu, mortality, mortality rate in sepsis, renal failure, aki, general nephrology dialysis and transplantation

## Abstract

Purpose

In the intensive care unit (ICU), acute renal failure is mostly part of multiple organ dysfunction syndromes with mortality ranging from 28%-90%, continuous renal replacement therapy (CRRT) is the predominant mode of RRT used in ICU. The main objective of the study was to evaluate the outcomes in patients with acute kidney injury (AKI) on CRRT in the ICU.

Methods

A retrospective chart review was conducted for all ICU patients with acute renal failure on CRRT in a tertiary care teaching hospital. A subgroup analysis was conducted between 15 days in hospital survivors and non-survivors to look for predictors of survival for patients on CRRT.

Results

Two-hundred twenty-six patients underwent CRRT from January 2007 to December 2013. The overall in-hospital mortality was 84.1%. Fifty-six patients (24.77%) survived to the 15-day post-CRRT mark. Acute respiratory failure requiring mechanical ventilation was associated with significantly increased mortality; 89.2% vs. 97.6% (P=0.008), ICU length of stay was significantly longer in the survivor group than the nonsurvivor group. Median±IQR; {20±24 vs 6±7(P: <0.0001)} and so were the ventilator-associated days {16±24 vs 4±6.5 (P: <0.0001)} and duration of CRRT {4.5±5.5 vs 2±2.0(P: <0.0001)}. Patients who survived had a lower incidence of metabolic acidosis {44.6% vs 62.9% (P: 0. 016)} and uremic encephalopathy {12.5% vs 26.5%; (P: 0.031)} but a greater incidence of volume overload {28.6% vs 15.9% (P: 0.031)} as compared to the non-survivor. Acute Physiology And Chronic Health Evaluation II (APACHE II) scores were significantly higher in the non-survivor group (mean SD) 26.9±28.0 vs. 23.9±25.8 (P: 0.0136).

Conclusions

This observational study in patients undergoing CRRT in an ICU setting revealed that the overall mortality was 84.1%. Fluid overload as an indication of CRRT was associated with improved 15 days’ survival whereas higher APACHE II scores and the use of mechanical ventilation were associated with reduced 15 days’ survival.

## Introduction

An acute kidney injury (AKI) requiring intermittent or continuous renal replacement therapy (RRT) significantly affects morbidity and mortality in critically ill patients and constitutes a substantial health care burden [1]. The mortality and morbidity associated with AKI remain to be a concern despite numerous improvements in RRT techniques and after significant advances in supportive intensive care unit (ICU) care. Importantly, the development of AKI constitutes an independent risk factor for death in the ICU.

Continuous RRT (CRRT) is widely used in ICUs and is often viewed as the superior approach in critically ill patients [1-4]. CRRTs refer to either dialysis (diffusion-based solute removal) or filtration (convection-based solute and water removal) treatments that operate continuously. The major advantage of continuous therapy is the slower rate of solute or fluid removal per unit of time. Thus, it has significant utility in hemodynamically unstable patients who cannot tolerate conventional intermittent hemodialysis [5]. This modality had theoretical advantages over intermittent renal replacement therapy, which are related to hemodynamic stability, optimal metabolic control, and fluid balance allowing nutritional supplementation. In ICU settings, AKI is mostly part of multiple organ dysfunction syndromes, and mortality in these patients ranges from 28%-90% [6-10].

Despite considerable advances in the management of critically ill patients and RRT, the high mortality rate in AKI could be explained, in part, by the individual characteristics of the patient population undergoing RRT. This may include a higher proportion of elderly patients, an increase in the severity and number of failing organs, and the delayed occurrence of AKI in patients whose survival is prolonged. However, despite the availability of CRRTs, the mortality associated with AKI in ICU patients on RRT remains high. So far, an insufficient number of studies have been conducted to identify the variable and characteristics that define the severity of illness and predict mortality in ICU patients on CRRT [11].

To better understand the characteristics of the patients undergoing CRRT, we conducted a study with the aim to establish an association between demographic characteristics and variables that define the severity of illness and the in-hospital and short-term mortality outcomes. We also identified a subgroup of patients that may benefit from CRRT regarding survival and quality of life post CRRT.

## Materials and methods

A single-center retrospective chart review was conducted on all adult patients above the age of 18 years undergoing CRRT at Staten Island University Hospital during the years 2007 to 2013.

Indications for initiating CRRT were fluid overload refractory to diuretics, hyperkalemia (serum potassium concentration >6.5 mEq/L), or rapidly rising potassium levels, refractory to medical therapy, severe metabolic acidosis (pH <7.1) in patients in whom the administration of bicarbonate was not indicated, such as those with volume overload, or those with lactic acidosis or ketoacidosis, in whom bicarbonate administration was ineffective and signs of uremia such as pericarditis, encephalopathy, or an otherwise unexplained decline in mental status. Drug Intoxication with hemodynamic instability was also used as indications for CRRT.

Exclusion criteria

Patients who could not tolerate CRRT for more than two hours were excluded from the study.

Study oversight and confidentiality

Each subject was assigned a unique consecutive number, not derived from any patient identifiers, known as the "code number." The code numbers were listed in a Master Subject Log and served to link the code number with their name, date of birth, and medical record number. RedCap (Vanderbilt University, Nashville, Tennessee) was used to enter all data. As it was a retrospective study with no intervention, a waiver of informed consent was submitted to the institutional review board (IRB). The study was subsequently approved by the IRB.

Study design

After including the eligible patients, their records were evaluated for various demographic and disease severity characteristics. The demographic characteristics assessed were age, sex, body mass index (BMI), and other medical co-morbidities, including coronary artery disease, heart failure, liver cirrhosis, hypertension, diabetes mellitus, end-stage renal disease (ESRD), and chronic obstructive pulmonary disease (COPD). Primary ICU diagnosis included the indication for ICU admission. Patients were also stratified based on acute respiratory failure requiring mechanical ventilation (noninvasive or invasive), number of pressors required, and the presence of sepsis. The length of ICU stay, indication for, and number of days on mechanical ventilation and CRRT were recorded for individual patients.

Endpoints

The primary endpoint included survival rate at 15 days after the termination of CRRT. Secondary endpoints included a three-month survival rate, new dialysis dependence post-discharge, worsening chronic kidney disease (CKD) from baseline, and length of ICU stay. APACHE II scores were calculated from the vital signs, oxygenation status, Glasgow coma scale (GCS), and laboratory parameters at the time of ICU admission. Lengths of ICU stay, indication for CRRT, and the number of days on mechanical ventilation and CRRT were recorded for individual patients.

Statistical analysis

Continuous variables were reported as the mean ± standard deviation (SD) and categorical variables as frequency and percentages. A chi-square test was done for categorical variables. A descriptive analysis was used to identify demographic data and clinical parameters. A subgroup analysis was performed between survivors and non-survivors to analyze the predictors of better outcomes with CRRT. All statistics were done using SAS software (SAS Institute, Cary, North Carolina). A P-value of <0.05 was considered significant and an independent predictor of better survival in patients on CRRT in the ICU.

## Results

Subject enrollment

A total of 258 patients underwent CRRT at our institution from January 2007 to December 2013; 21 patients were excluded, as they didn’t complete a minimum of two hours of CRRT. Two-hundred thirty-seven patients were included in the study, 11 patients had missing information related to demographics and outcomes, and hence only 226 patients were included in the final analysis.

Baseline characteristics

Among the 226 subjects n(%), 127 (56.0) were males. The mean age of the population was mean±SD 66.8±14.2. All 226 patients were in the Intensive care unit at the time of CRRT, and 43 (19) of them were postoperative surgical ICU patients. The majority of them had septic shock 164 (73) as their ICU diagnosis followed by acute respiratory failure150 (66) and gastrointestinal bleed 20 (9). See Table 1.

**Table 1:** Baseline characteristics of patients undergoing CRRT (N:226) N: sample size; n: number of patients; STEMI: ST-segment elevation myocardial infarction; NSTEMI: non-ST-segment elevation myocardial infarction; ICU: intensive care unit; CHF: congestive heart failure; GI: gastrointestinal; ACS: acute coronary syndrome; COPD: chronic obstructive pulmonary disease; CRRT: continuous renal replacement therapy

Baseline characteristics	
Male sex n(%)	127(56)
Female sex n(%)	99(44)
ICU diagnosis	n(%)
Acute respiratory failure	150(66)
Acute CHF	18(8)
Septic shock	164(73)
GI bleed/Hemorrhagic shock	20(9)
Arrhythmia	12(5)
ACS (STEMI/NSTEMI)	14(6)
Postop	43(19)
Intracranial bleed	3(1)
Baseline comorbidities	
Congestive heart failure	59(26)
End-stage renal disease	29(13)
Diabetes mellitus	90(40)
Hypertension	156(69)
Cirrhosis	20(9)
Coronary artery disease	79(35)
COPD	41(18)
Indications for CRRT	
Refractory hyperkalemia	31(14)
Metabolic acidosis (ph<7.2)	132(58)
Uremic encephalopathy/Pericarditis	53(23)
Drug intoxication	4(2)
Refractory fluid overload	43(19)

Twenty-nine out of 226 (13%) patients had ESRD at the time of presentation and were dialysis-dependent. Ninety-five percent of the patients required mechanical ventilation at the time of initiation of CRRT. The most common indication for RRT was severe metabolic acidosis 132 (58%) followed by uremic encephalopathy 53 (23%) and refractory fluid overload 43 (19%).

Primary outcome

Overall in-hospital mortality was 84.1% (190/226). Only 56 patients (24.77%) survived to the 15-day post-CRRT mark. Twenty out of 56 (35.71%) patients died during the same hospitalization. Among the 36 who survived, 16 (28.57%) were discharged home, 15 (26.7%) were discharged to a skilled nursing facility, and five (8.9%) were released to other destinations.

Secondary outcomes and subgroup analysis

A subgroup analysis between the 15 days' survivor and non-survivor groups was conducted. Acute respiratory failure requiring mechanical ventilation was associated with significantly increased mortality; 89.2% vs. 97.6% (p =0.008). Baseline comorbidities did not have a significant impact on 15-day survival. The ICU length of stay was significantly longer in the survivor group than the nonsurvivor group median±IQR {20±24 vs. 6±7 (P: <0.0001)} and so were the ventilator-associated days {16±24 vs. 4±6.5 (P: <0.0001)} and duration of CRRT {4.5±5.5 vs. 2±2.0(P: <0.0001)}. See Tables 2-3.

**Table 2:** Risk factors associated with mortality among patients undergoing CRRT (N=226) N: sample size; n: number of patients; MV: mechanical ventilation; P<0.05 considered being significant; CHF: congestive heart failure; CRRT: continuous renal replacement therapy

15-day survival	Survived	Didn’t survive	P-value
Sample size	56	170	
Age mean+/-SD	64.3±16.0	67.7±13.6	0.058
Male n (%)	26(46.4)	101(59.4)	0.089
Female n (%)	30(53.6)	69(40.6)	0.089
Septic shock	41(73.2)	123(72.3)	0.973
Mechanical ventilation	50(89.2)	165(97.6)	0.008
Acute CHF	6(10.7)	11(6.5)	0.379
Gastrointestinal bleed	5(8.9)	15(8.8)	0.981
Postoperative renal failure	13(23.2)	30(17.7)	0.432
Intra-cranial bleed	0(0.0)	3(1.8)	0.422
Acute coronary syndrome	3(5.4)	11(6.5)	1.000
Arrhythmias	6(10.7)	6(3.5)	0.034
Indication n(%)			
Hyperkalemia	6(10.7)	25(14.7)	0.451
Metabolic acidosis	25(44.6)	107(62.9)	0.016
Fluid overload	16(28.6)	27(15.9)	0.031
Uremic (Encephalopathy/Pericarditis)	7(12.5)	45(26.5)	0.031
Drug intoxication	2(3.6)	2(1.2)	0.257

**Table 3:** Subgroup analysis of patients on CRRT between survivors and non-survivors APACHE II: Acute Physiology And Chronic Health Evaluation II (acute physiology score + age points + chronic health points); CRRT: continuous renal replacement therapy

15-day survival	Survived	Didn’t survive	P-value
Days on vent Median±IQR	16±24	4±6.5	<0.0001
CRRT duration (days) Median±IQR	4.5±5.5	2±2.0	<0.0001
ICU duration Median±IQR	20±24	6±7	<0.0001
Lactate Median±IQR	2.8 ±4.1	6±8.2	<0.0001
Platelets Median±IQR	150±148	120±134	0.0374
Albumin Median±IQR	1.9±0.8	1.8±0.8	0.0164
Creatinine Median±IQR	3.7±2.5	2.7±2.7	0.5510
No of vasopressors ≥2:n(%)	36(64.3)	137(81.5)	0.037
APACHE II scores (Mean±SD)	23.9±25.8	26.9±28.0	0.0136

Diagnosis at the time of intensive care unit admission did not have a significant impact on 15-day survival among patients undergoing CRRT. Patients who survived had a lower incidence of metabolic acidosis {44.6% vs 62.9% (P: 0.016)}, uremic encephalopathy{12.5% vs 26.5%; (P: 0.031)} but a greater incidence of volume overload {28.6% vs 15.9% (P: 0.031)} as compared to the non-survivor. No significant difference was found for hyperkalemia {10.7% vs 14.7% (P: 0.451)} between the two groups.

Patients who didn’t survive for 15 days had elevated APACHE II scores, elevated lactate, low platelets, low albumin, and higher use of vasopressors as compared to those who survive. There was no significant difference between the creatinine levels of the two groups (Table 3).

## Discussion

CRRT has become the choice of RRT for the treatment of AKI in patients admitted to ICUs. In this single-center retrospective review, the overall in-hospital mortality of patients undergoing CRRT in ICU was found to be 84.2%, with only 24.8% of patients surviving the 15-day post-CRRT mark. The reported mortality in patients undergoing CRRT in the ICU ranges from 37% to 75% [12-13]. Observed mortality in our study is higher than the reported death, probably due to the overall morbidity of our population observed by the fact that the majority of our patients (95.5%) were on mechanical ventilation and 76.5 % of patients were on more than or equal to two vasopressors at the time of initiation of CRRT. Moreover, these two factors were independently associated with increased mortality after 15 days. Furthermore, the majority of our patients were non-surgical ICU patients, with merely 19% of postoperative subjects in our study, and the sample size was too small to show any significant survival difference in this sub-group. A recent study published in JAMA in surgical ICU patients showed a mortality rate of 53.7% for patients undergoing CRRT in ICU [14].

Unlike other studies, the patients who survived the first 15 days received a longer duration of CRRT as compared to those who did not survive [14]. This may just reflect that patients who showed signs of recovery while being on CRRT were continued for longer durations before switching to regular hemodialysis.

Outcomes were unaffected by age and gender while some previous studies have associated advancing age and male sex with adverse outcomes. Acute respiratory failure requiring mechanical ventilation was associated with significantly increased mortality. As expected, high baseline APACHE II scores, low albumin, and elevated lactate levels were associated with increased mortality, which also reiterates findings from multiple previous studies [15-16]. In another study, the Liano score has been reported to identify a group of patients on dialysis for acute renal failure with a near 100% chance of mortality but did not show discrimination between those patients who died in hospital and those who did not [8,17]: Douma and associates showed in their report that the use of both the APACHE and Liano scores would be better at predicting hospital mortality [8].

Interestingly, baseline comorbidities did not have a significant impact on the 15-day mortality rate. Septic shock was the most common ICU diagnosis, and hyperkalemia was the most common indication for CRRT. Severe metabolic acidosis with Ph <7.2 was associated with significantly increased mortality as compared to other indications for CRRT, whereas fluid overload as an indication for CRRT had better 15-day survival in our study. This could be explained by the fact that CRRT is best known for its fluid balance ability and patients with fluid overload as an indication for dialysis may have better outcomes as compared to other indications for dialysis.

In conjunction with other studies, our study also aims at determining the variables that may predict the short-term mortality rates in critically ill patients on CRRT. This, in turn, would help in identifying the patient population that would most likely benefit from this expensive treatment modality that may otherwise be a futile exercise in other patient populations, thereby controlling healthcare expenditure. However, predictors may vary with different subjects, depending upon the underlying condition. For example, in patients after cardiopulmonary bypass, lactate levels may rise regardless of the patient’s condition or the presence of peripheral circulation insufficiency. In these patients, the cardiac index or presence of a low output syndrome will be a better predictor than the lactate levels.

It should also be noted that our retrospective chart review might not be used to determine a cause-effect relationship. Furthermore, large-scale multicenter studies with larger sample sizes are needed to evaluate the predictors better to set standards while employing CRRT.

## Conclusions

In conclusion, the requirement of CRRT in ICU patients may portend a poor prognosis, as it may carry a very high risk of mortality of up to 84.1%, as shown in our study population. Fluid overload as an indication of CRRT appears to be a better predictor of survival in patients undergoing CRRT. Patients with significant medical illness indicated by higher APACHE II scores may have worse outcomes and, therefore, the appropriate selection of patients for this procedure is of paramount importance, not only to improve survival but also for the efficient utilization of health care resources. Besides, this is a single-center observational study and, perhaps, the results cannot be extrapolated for the general population of the United States. However, more robust, multicenter randomized controlled clinical trials are needed to further evaluate the role of CRRT in patients with acute renal failure in the ICU.
